# A systematic trust management system for RPL based IoT networks using machine learning

**DOI:** 10.1038/s41598-026-47585-5

**Published:** 2026-07-07

**Authors:** Himani Tyagi, Rajendra Kumar, Santosh Kr Pandey

**Affiliations:** 1 Jamia Millia Islamia University, New Delhi, India; 2 Ministry of Electronics and Information Technology, New Delhi, India

**Keywords:** Trust, Management system, Routing attacks, Detection, KELM (Kernel Extreme Learning Machine), Machine learning, Clustering, Engineering, Mathematics and computing

## Abstract

As IoT networks continue to evolve, concerns about their security risks are growing. Several security gaps still exist within IoT systems like Blackhole, Decreased Rank, Version Number, and Flooding attacks. During these attacks the intruder cheat legitimate nodes. Hence, affecting trustworthiness. One potential solution is the Trust Management System (TMS). TMS evaluates the trustworthiness of nodes, offers real-time predictions, detects attacks and aids decision-making to offer secure routing. This work proposes a lightweight, reliable and dynamic TMS to enhance IoT network security. There are two primary challenges in developing an AI-driven security system for IoT. The first is the availability of IoT datasets that include trust-based attacks and trust value as labels. The second is the inherent uncertainty of trust, which complicates the development of a dynamic system. The proposed TMS relies on three novel set of trust indicators: network flows, recommendations, and social behaviour. These indicators are aggregated using a modified Beta Distribution function to represent trust as labels in the dataset. The work also discusses the design of a lightweight and real-time trustworthiness predictor based on Kernel Extreme Learning Machine (KELM), which uses the labels from Beta Distribution for more accurate and reliable predictions. Furthermore, a dynamic threshold evaluation module is introduced to classify the nodes as either trustworthy or untrustworthy based on distance metrics. The proposed TMS is validated against four types of trust-based attacks—Blackhole, Decreased Rank, Version Number, and Flooding—which have not been adequately addressed in existing literature. The performance of proposed model is also validated on binary and multiclassification. Comparisons existing linear and non-linear machine learning models demonstrate that the proposed system outperforms current state-of-the-art TMS solutions, achieving 99.95% accuracy, 99.9% precision, 99.96% recall, and a minimal misclassification rate of just 0.05%.

## Introduction

The Internet of Things (IoT) has sparked a technological revolution in automation and robotics, drawing significant attention from researchers and scientists focused on developing smart solutions. These solutions manifest in various areas, including smart vehicular systems, smart agriculture, smart cities, smart healthcare, and smart manufacturing^1^. At the heart of these smart solutions are sensor devices integrated with data-sharing capabilities. The exchange of sensitive data between devices occurs before it reaches its intended destination, facilitated by routing protocols. However, widely used protocols like Adhoc On-Demand Distance Vector (AODV), Dynamic Source Routing, and Open Shortest Path First (OSPF) have been found inefficient for low-power, lossy networks like IoT^2^. As a result, a specialized routing protocol for IoT, called RPL, has been introduced. RPL is tailored for low-power, lossy networks and is specifically designed to provide lightweight and efficient routing in IoT-based 6LoWPAN (IPv6 over Low-power Wireless Personal Area Networks). RPL has gained significant popularity in both academic and industrial circles due to: its ability to deliver effective routing for resource-constrained, smart, IP-enabled IoT nodes, its adaptability to various network topologies, and its support for Quality of Service (QoS)^3^ A key feature of RPL is the use of a Destination Oriented Directed Acyclic Graph (DODAG). The root node of this graph serves as a gateway to which all other nodes are connected. The nodes are responsible for sensing data from the physical world and using the gateway to transmit the sensed data to the destination. This protocol is open, self-organizing, self-healing (in case of node failure), and designed for resource-constrained environments. However, its openness makes it susceptible to various threats and attacks, with trust-based attacks being among the most common^4^. These are also known as routing or internal attacks. Trust-based attacks compromise the trustworthiness of other nodes by providing inaccurate information about their position, status, and distance from the root node or by flooding the destination with unnecessary packets^5^. These attacks lead to resource exploitation, incorrect information dissemination, faulty neighbour selection, disrupting the RPL topology, data leakage, and denial of legitimate services^6^. The successful, trustworthy communication during the routing process improves PDR (packet delivery ratio) and enhances overall network performance. Therefore, there is a pressing need for solutions that prevent such attacks.

Trust Management Systems (TMS) are frequently discussed in the literature as an effective solution for securing IoT networks and preventing routing attacks^7^. TMS are responsible for evaluating the trustworthiness of nodes, predicting future trustworthiness, and isolating nodes deemed less trustworthy. This system provides essential behavioural guidelines and protection during entity interactions^8^. However, trust is inherently subjective, highly dynamic, and uncertain, making its definition and measurement complex. To effectively measure trust, traditional and limited trust parameters are considered in the literature^6,9^. However, an additional set of potent indicators is necessary to comprehend trust. Therefore, this study introduces indicators that incorporate interaction-based (direct and indirect) metrics and social factors that are not considered in the literature. Further, existing methods, such as linear combinations or weighted summations of features, are often used to aggregate trust values^8,10^. These methods are biased, inefficient, and fragile, particularly because trust values are dynamic and determining the right weights is challenging. Another limitation of the existing system is the frequent use of model driven approaches like fuzzy logic, Bayesian networks and Dempster–Shafer theory^7,8,10^ which depends on domain knowledge for decision-making. In contrast, data-driven approaches like machine learning uses statistical analysis to assess trust^9,10^. Additionally, machine learning-based solutions offer protection and mitigation of zero-day attacks or any outlier situation that has not been before^6,19^. Therefore, in an increasingly interconnected digital environment like IoT, Machine Learning (ML) is a valuable tool for improving trust assessment and management by continuously refining and adapting trust indicators based on real-time data^6,7^. Furthermore, an ontology provides a systematic approach involving several key steps, such as trust aggregation, trust evaluation, real-time trust prediction, and attack detection. However, this is often lacking in the literature. As a result, there is a clear need for a trust ontology in the Internet of Things^8^. Additionally, in recent studies, attack detection has been treated as a binary classification or anomaly detection problem. On the other hand, in an IoT system, multiple attacks are possible. Therefore, the proposed model is designed with IoT limitations in mind, particularly constraints on computational complexity, memory usage, energy consumption, and communication overhead.

Therefore, the main objectives of this study are as follows:

I. To integrate direct and indirect behaviours with socialness as a novel set of trust indicators to deal with uncertain device behaviours using modified beta distribution. Therefore, relies on lightweight feature extraction.
II. To evaluate trustworthiness of each node using a modified beta distribution function, which incorporates a balanced set of good and bad behaviours. This approach addresses the subjectivity often introduced by traditional weighted summation methods. Further, a lightweight trust prediction module using KELM is also introduced.
III. To propose a systematic Trust Management System (TMS) consisting of four module pipeline: a Trust indicator selection module, a Trust aggregator module, a Trust Prediction module and an attack detection module. This framework aims to accurately identify four types of trust-based attacks (routing attacks) such as Flooding, Blackhole, Decreased Rank, and Version Number and isolate less trustworthy nodes using machine learning techniques. These four types of attacks are rarely addressed in the existing TMS literature.
IV. To compare the performance of proposed TMS for both binary and multiclass classification tasks. Additionally, a novel stacked model is introduced for handling multiclass classification problem.

The remaining paper is organized as follows: “Related work” outlines the existing TMS in IoT networks. “Proposed data driven trust management system” presents the proposed TMS explaining step by step functionality of each module. The experimental results of the proposed system are shown in “Experimentation and results”. The performance comparison of proposed TMS with existing techniques is outlined in “Comparison with state of the arts techniques”. “Conclusion” concludes our paper and presents suggestions for future directions.

## Related work

Due to security vulnerabilities in the RPL protocol, it is susceptible to various routing attacks that are difficult to detect using traditional security frameworks. As a result, this section discusses recent studies on Trust based solutions for routing attacks in IoT.

Ahmadi and Javidan^6^ implemented a trust-based attack detection model for RPL-based IoT networks using a deep learning based LSTM model. In this system, the packet forwarding ratio (PFR) serves as the key trust indicator to distinguish between a normal and malicious nodes. The proposed system critically analyse the nodes behaviour during attacks and normal scenarios at different time spans. Inspired from^16^ the model utilizes the past three behaviours of a node to forecast its future behaviour. The resulting difference between actual and expected behaviour is used to calculate a trust score. The effectiveness of the proposed model is validated under blackhole and selective forwarding routing attacks.

Alghofaili et al.^7^ proposed a Trust Management System using multi-criteria decision-making (SMART) approach and LSTM. The model is proposed by incorporating three trust indicators packet loss, delay, and throughput. These indicators are aggregated using Shannon entropy. Afterwards, a threshold-based labelling is applied, and LSTM (Long Short-Term Memory) is used to classify any instances of misbehaviour. The model demonstrates promising results, achieving an accuracy range of 98.33–99.97%. However, the model has not been validated against specific routing attack types, but a simple misbehaviour detection was performed.

Jaysinghe et al.^10^ proposed a trust computational model using machine learning techniques. The work focuses on various social features such as reputation, experience, and knowledge to evaluate the trustworthiness of nodes. This model is specifically designed for Social IoT (SIoT) networks. The study highlighted the significance of social attributes in evaluating the correct behaviour. These attributes are integrated using a weighted summation method. The system then employs KMeans clustering to classify samples as trustworthy or untrustworthy within the dataset. A Support Vector Machine (SVM) classifier is utilized to categorize transactions as trustworthy or untrustworthy based on the assigned labels. The proposed model achieved a precision of 89%, recall of 100%, FPR of 0, FNR of 0%, and TNR of 0.995.

Ma et al.^16^ implemented a Trust prediction model based on deep learning. For this Long Short Term Memory (LSTM) model is employed to predict a node’s next behaviour by analysing the past three behaviours. The difference between the actual and predicted behaviour is used as a trust value, which is then factored into decision-making. The model shows an accuracy of 98.3%, MSE 0.005 and R^2^ of 0.88. This model relies solely on network flows for its analysis and does not include any social features to enhance decision accuracy and precision.

Muzammal et al.^17^ suggested a trust-based secure protocol for IoT routing attacks in RPL. The proposed algorithm evaluates weighted summation of various trust parameters, including location, recommendation, and network flow. The SMTrust model, however, demonstrates lower energy consumption in nodes when calculating trust values. Evaluating trust through weighted values presents a challenge, as it relies on subjective judgment and expertise. Moreover, the threshold for identifying malicious nodes is determined manually based on experience, suggesting the need for a statistical method to enhance this process.

Sharma et al.^18^ proposed BDTrust, a trust management system for the detection of on off attacks in RPL based IoT networks, where the authors combine behavioural (success rate, satisfaction level, credibility) indicators and data (data deviation) indicators to compute the final trust value. Trust aggregation is performed using a proposed logarithmic function. Here, a trust update module is introduced to attenuate the presence of malicious nodes in IoT networks. The proposed model demonstrated high accuracy and low precision.

Djedjig et al.^19^ developed a trust based security system for detecting rank and blackhole attacks in a RPL based IoT network. The proposed method combines selfishness, honesty, and energy to evaluate the direct trust of each node. The trust score added to DIO messages is used by a node while evaluating a parent. The objective of the proposed system is to isolate the nodes with low energy and high packet delivery ratio (PDR). Further, Jiang and Liu^5^ proposed a lightweight trust management system for detecting selective forwarding attack in IoT. The proposed model used only one behaviour: packet forwarding rate (PFR) to evaluate trust of nodes. The trust score is represented by deviation from the PFR’s mean. The proposed system showed high FAR of 0.4 and low AUC of 75.8%.

Caminha et al.^20^ proposed a smart trust management system for detecting On-off attack in IoT networks. The proposed system analysed temperature data from Aarhus, Denmark, over the course of one month, consisting of 4111 samples. The deviation from the average temperature is considered to evaluate a node’s trustworthiness. The out of range value is considered as probability of being misbehaviour, then the behaviour of that node is evaluated on longer time windows. The time window is evaluated using decision function. The proposed system uses support vector machine (SVM) based hyperplane creation to classify nodes in normal, broken and on off category. The model is validated on real rime dataset, which shows 96% precision, 0.85 recall and 0.87 F1 score.

Yu et al.^21^ have designed a trust management scheme in IoT by employing entropy based trust aggregation and Dempster Sheffer theory (DS theory) based trust evaluation named ETES. The model relies on data packet consistency, packet forwarding capacity, repetition rate, delay, and integrity to evaluate the trust of a node. These indicators are combined using the entropy method to deal with the device’s uncertainty. DS theory is employed in the system to evaluate trust. The simulation of the proposed system is done using MATLAB simulator. The system can detect five types of routing attacks like DoS, data forgery, On Off, bad mouthing and selective forwarding. Feng et al.^22^ have also employed Dempster Sheffer theory, to design a trust management scheme for IoT networks. The system employs direct trust and indirect trust to evaluate the overall trust of a node. The system used only network flow based trust indicators like Received packets rate RPF, successfully sensing packet rate SPF, Packet forwarding rate and Node availability HPF to compute trust values of nodes. The proposed system shows promising results during on off and bad mouthing attacks.

Wang et al.^23^ have proposed a context aware trust management system for Internet of Vehicular (IoV) System. The proposed system relies on direct, indirect and context trust indicators. The performance of proposed model is validated against rural and urban scenarios. The method uses KMeans clustering for labelling each sample and uses these labels to give input to KNN classifier for appropriate classification of trustworthy and untrustworthy transactions. Again, the method doesn’t incorporate trust-based attacks in the evaluation and classification.

Additionally, a summary of the related works, including its advantages and limitations, is presented in Table 1.

**Table 1:** Summary of trust based solutions in IoT.

References	Trust indicators	Attacks addressed	Advantages	Type of detection	Limitations	Steps covered in proposed system
^5^	Packet forwarding ratio	Selective forwarding attack	The model shows good performance with low FAR	Binary	A single trust indicator is used. Trust ontology is missing	TE=√ TA=× TP=× AD=√
^6^	PFR	Blackhole and selective forwarding	The proposed model shows a close relation between predicted and actual behaviour	Binary	Only a single trust indicator is used. Covering limited number of RPL specific attacks	TE=√ TA=× TP=√ AD=√
^7^	Three network flow based trust indicators	Misbehaviours are considered	Having a high accuracy of 99.87% in attack detection	Binary	Limited number of trust indicators. Routing attacks are not considered	TE=√ TA=√ TP=√ AD=√
^10^	Social features based trust indicators	Misbehaviours are considered	Good performance in terms of TPR, Recall, FPR, FNR and TNR	Binary	The model’s performance was assessed only for binary classification, and the dataset contained limited samples. Therefore, the precision of the model is low. No consideration of routing attacks	TE=√ TA=√ TP=× AD=×
^17^	Direct, indirect and location based trust indicators	Rank and blackhole	The model shows promising results regarding PDR, energy consumption and throughput	Binary	The trust aggregation relies on subjective judgment and expertise	TE=√ TA=√ TP=√ AD=√
^18^	Direct, indirect and data trust indicators	On-off attacks	The system shows fast trust convergence rate	Binary	Limited number of attacks are considered	TE=√ TA=√ TP=√ AD=√
^19^	Social features like honesty, selfishness and energy	Rank and blackhole attacks	The objective is to detect and isolate nodes with less energy and having high PFR	Binary	Limited number of routing attacks are considered. Trust ontology is missing	TE=√ TA=× TP=× AD=√
^20^	Temperature recordings	On off attack	The trust model show high accuracy	Binary	Single trust indicator is used. Binary classification is done. The performance is validated on a limited dataset	TE=√ TA=× TP=√ AD=√
^21^	Direct and indirect	Data forgery, Selective forwarding, On off, DoS and Bad mouthing	Trust aggregation is done by combining network flow features to detect attacks.DS theory is employed to finally evaluate the trust	Binary	The model show low recall and precision	TE=√ TA=√ TP=× AD=√
^22^	Direct and indirect	On off attacks and bad mouthing attack	The trust model uses DS theory and show high accuracy in attack detection	Binary	Mass function evaluation is a challenging task using DS theory	TE=√ TA=√ TP=× AD=√
^16^	14 Network flow based trust indicators	Mirai	The trust model predictions show low MSE, high accuracy and R^2^	Prediction model	Attack detection is not proposed. No consideration of routing attacks. Trust ontology is missing	TE=√ TA=× TP=√ AD=×
^23^	Direct, indirect and context based trust indicators	Incorrect information and misbehaviour	High accuracy and precision	Binary	Very simple model to aggregate trust. No trust based attack is considered. Attack detection is done using KNN classifier, which is cost-intensive	TE=√ TA=√ TP=× AD=√
Our proposed	Direct, indirect and socialness	Version number, flooding, rank, blackhole attack	A lightweight, dynamic and real time trust predictor and attack detector is proposed	Binary and multiclassification	Four types of RPL based attacks are considered. Other attacks are to be included	TE=√ TA=√ TP=√ AD=√

RPL, Routing protocol for low power and lossy networks; FAR, False Alarm Rate; KNN, K nearest neighbour; TPR, True Positive Rate; FPR, False positive rate; FNR, False Negative Rate; DS, Dempster Shaffer; TE, Trust evaluation; TA, Trust aggregation; TP, Trust prediction; AD: Attack detection; PFR, Packet Forwarding Rate; PDR, Packet Delivery Ratio.

## Proposed data driven trust management system

**Fig. 1:**
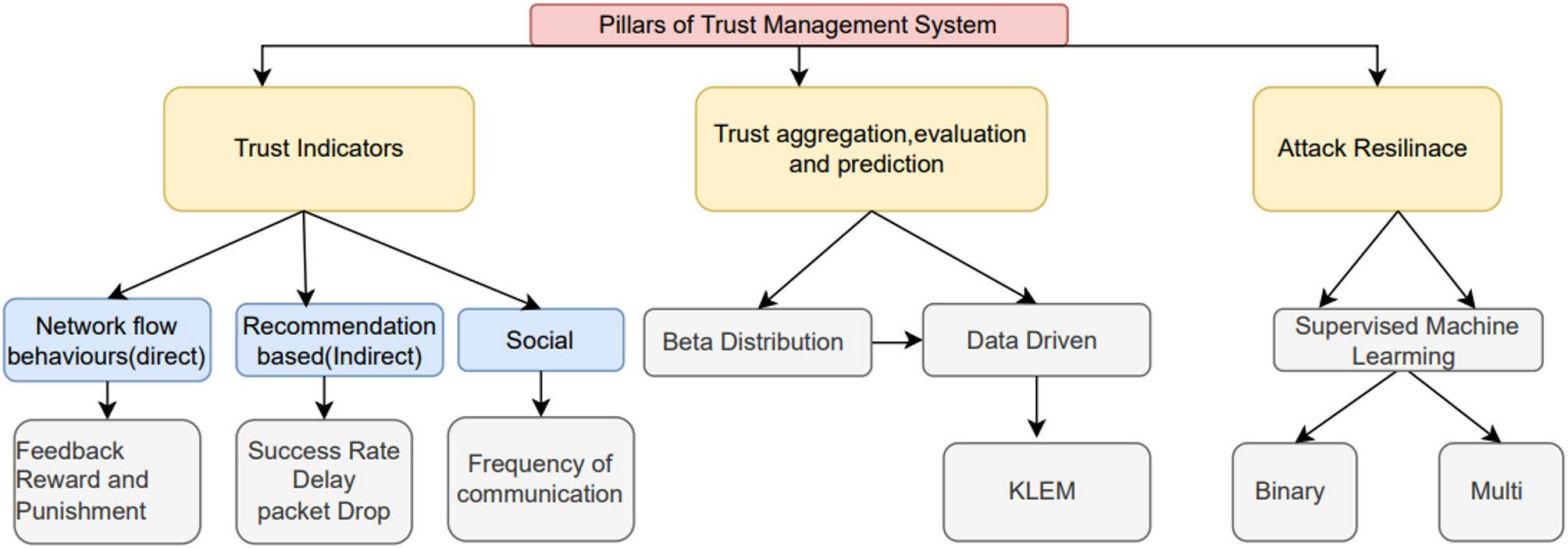
Three main pillars of the proposed TMS.

**Fig. 2:**
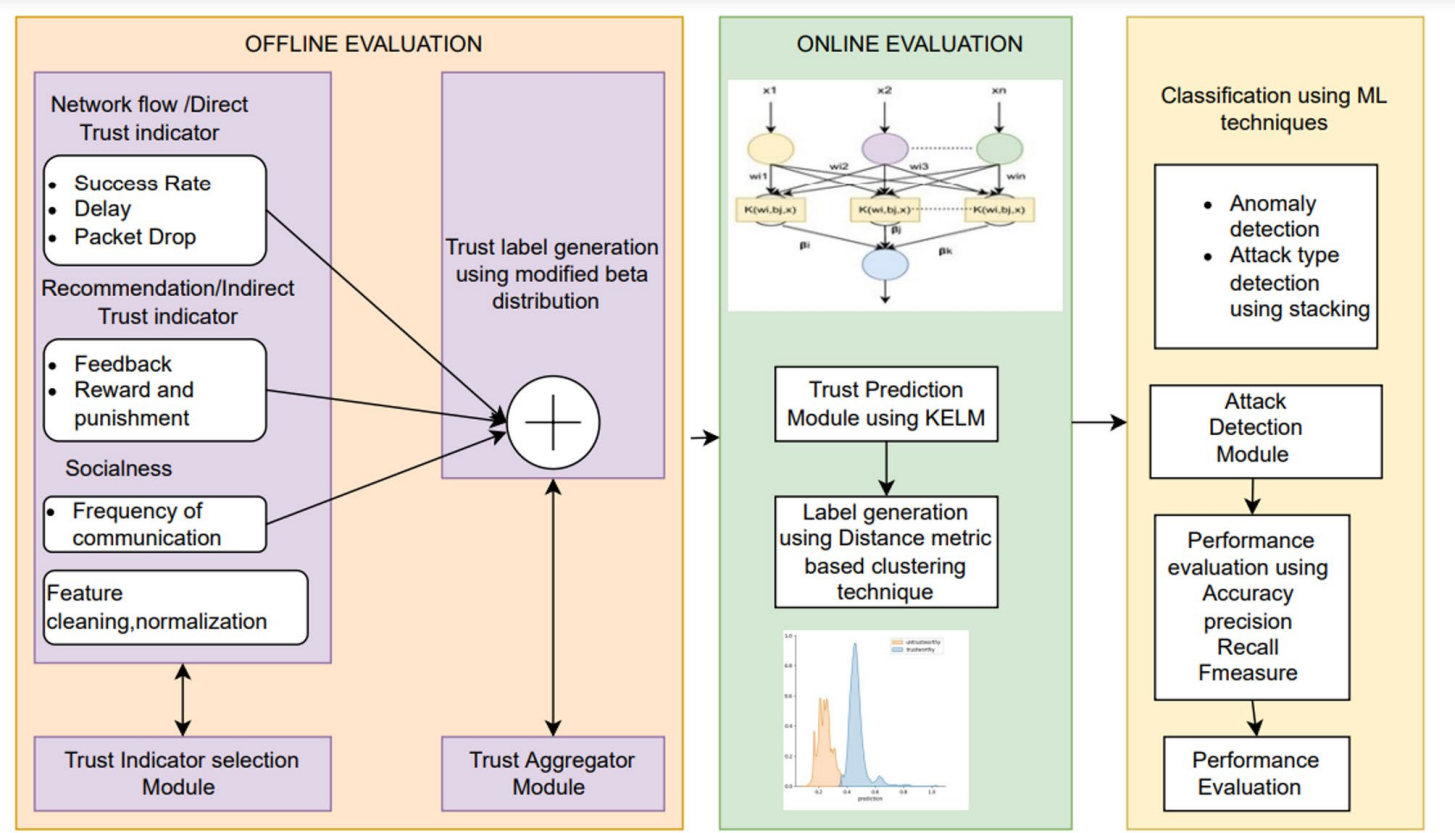
Working pipeline of proposed TMS.

Trust Management Systems (TMS) are widely discussed in security literature concerning Wireless Sensor Networks (WSN). The proposed system works in a pipeline consisting of four key modules: a trust indicators selection module, a trust aggregation module, trust prediction module, and an attack detection module. The trust indicator module selects a balanced set of three indicators: direct (network flows), indirect (recommendations) and social behaviour, which are further classified as shown in Fig. 1. The importance of these indicators is clearly visible in^10,13,17,23^. The trust aggregation module combines the aforementioned trust indicators and assesses the trust level for each transaction using modified beta distribution. Further, the trust prediction module using Algorithm 1 forecasts each node’s trustworthiness to guide the final decision regarding their behaviour employing lightweight KELM. Further, the trust values as Labels are generated using Algorithm 2 and the output is given to attack detection module for real time binary and multiclassification using the forecasted values. A detailed explanation of the functionality of each module of the proposed TMS is shown in Fig. 2.

Step 1: trust indicators selection module.

I. Network flow based (direct):

- Delay: Delay is defined as the difference between the average time a node takes to send a packet and the average time it takes to receive the packet. From the given features, this is evaluated using Eq. (1):

1 $$\:{Delay}_{ij}={Transmission\:average\:time}_{ij}-{Reception\:average\:time}_{ij}$$

- Packet drop rate (PDR): it is defined as the ration of number of packets received from the source to number of packets sent from the source, it is expressed using Eq. (2):

2 $$\:{packet\:drop\:rate}_{ij}=\frac{{reception\:count}_{ij}}{{transmission\:count}_{ij}\:}$$

- Success rate (SR): It is defined as the number of successful and unsuccessful transactions over a period of time. This is a powerful tool in representing the behaviour of a trustee. This is evaluated using an elastic windowing method. It is evaluated using Eq. (3):

3 $$\:SR\:i,j,t=\frac{{NS}_{i,j,t}}{{NS}_{i,j,t}+{NU}_{i,j,t}}$$

where NS is the number of successful transactions (Labels as 0) between i and j in time unit t, NU is the number of unsuccessful transitions (Labels as 1) between i and j in time unit t. $\:t{ransmission\:count}_{ij}$

II. Recommendation based (indirect):

- Reward and punishment (RP): It is defined as the rewards and punishments disseminated to trustor j by trustee i for delivering a service successfully or unsuccessfully. This is evaluated with the help of Packet Drop Rate (PDR) using Eq. (4):

4 $$\:RPi,j,t=\:{PDR}_{i,j,t}{\:e-}^{\frac{NS\:i,j,t}{NS\:i,j,t+NU\:i,j,t}\:}$$

- Feedback: It is defined as the recommendation based on the service offered by the trustee node to the trustor. This factor is inspired from the social sciences as after a service the feedbacks based on the satisfaction level of a entity are given in the range of 0 star being utmost dissatisfy and 5 being highly satisfied. Based on this, the feedbacks are evaluated using a threshold method as shown in Eq. (5).

5 $$\left. {F{B_{i,j,t}}} \right\}=\begin{array}{*{20}{c}} {1,} \\ {0.5,} \\ {0,} \end{array}\begin{array}{*{20}{c}} {if\,\,SRi,j,t=1} \\ {\,\,\,\,\,\,\,\,if\,=1<SR~i,j,t<0.3} \\ {if\,SR~i,j,t<0.3} \end{array}$$

The thresholds here are decided by critically analyzing the data during attack and normal traffic.

III. Socialness based:

- Frequency of communication: This feature is defined as the number of past communications the trustor has with the trustee. The more communication there is, the more credibility for the trustor node. This is evaluated using Eq. (6).

6 $$\:{N}_{ij}=counting\:the\:EquationNumber\:of\:transactions\:between\:trutor\:i\:and\:trutee\:j\:$$

Step 1.1: Data preprocessing.

After all the features are extracted, a series of data cleaning, exploratory data analysis (EDA) and normalization are performed. The presence of null or NAN values creates trouble in understanding the hidden patterns in the data for the ML model. Hence, when these NAN values are replaced with 0 these were depicting divide by zero values error. These values are available for all the places in the dataset where the denominator is zero after calculation and hence are replaced with 0 to remove ambiguity and complexity. Then, deep Exploratory Data Analysis (EDA) is performed to identify hidden patterns, missing values, or outliers, and to analyze statistical measures such as the mean, median, correlation, standard deviation, and visualizations. To ensure a smooth and efficient machine learning process, all feature values are scaled to a range between 0 and 1 using the *MinMax* scaling function under sklearn library. The process is shown in Eq. (7).

7 $$\:Normalised\:value\left({X}_{i}\right)=\frac{{z}_{i}-\mathrm{m}\mathrm{i}\mathrm{n}\left(z\right)}{\mathrm{max}\left(z\right)-\mathrm{m}\mathrm{i}\mathrm{n}\left(z\right)}$$

Step 2: Trust aggregator module.

This module combines direct, indirect, and social trust indicators between sensor nodes i and j using a prior probability distribution, which is popular as beta distribution. The Beta distribution provides a continuous prior or model for the probability of occurrence of an event in a binomial distribution. The distribution is flexible, robust and effective in representing the behaviour of a node considering various good and bad behaviours. In this work, the local trust value is evaluated considering six fundamental parameters as delay (bad behaviour), Packet drop rate (bad behaviour), Success rate (good behaviour), feedback (good behaviour), Reward and punishments (good behaviour), Frequency of communication(N) (bad behaviour). Therefore, the calculation of the Beta parameter is designed to average the impact of each condition, resulting in a value that remains stable and does not vary with the specific values of the input variables. The basic equation of a beta distribution looks like:

8 $$\:{Behaviour}_{ij}=E(f\left(p;{\upalpha\:}\mathrm{i}\mathrm{j}\:+1,{\upbeta\:}\mathrm{i}\mathrm{j}+1\right)=\frac{{\upalpha\:}\mathrm{i}\mathrm{j}\:+1}{{\upalpha\:}\mathrm{i}\mathrm{j}\:+{\upbeta\:}\mathrm{i}\mathrm{j}+2}$$

where, $\beta ij$ represents values for good behaviours between node i and j, whereas $\beta ij$ are the values of bad behaviours.

The modified above equation for our problem is represented as:

9 $$\:local\:trust\:value=E(f\left(p;{\upalpha\:}\mathrm{i}\mathrm{j}\:+1,{\upbeta\:}\mathrm{i}\mathrm{j}+1\right)=\frac{Success\:rate+feedback+Reward\:and\:punishment+1}{Success\:rate+feedback+Reward\:and\:punishment+delay+packetdrop+\left(\frac{1}{N}\right)+2}$$

where,

$$\begin{gathered} f\left( {p;\alpha ,\beta } \right)=\frac{{{p^{\alpha - 1}}{{\left( {1 - p} \right)}^{\beta - 1}}}}{{\mathop \smallint \nolimits_{0}^{1} {u^{\alpha - 1}}{{\left( {1 - u} \right)}^{\beta - 1}}du}} \hfill \\ =\frac{{\Gamma \left( {\alpha +\beta } \right)}}{{\Gamma \left( \alpha \right)\Gamma \left( \beta \right)}}{p^{\alpha - 1}}{\left( {1 - p} \right)^{\beta - 1}},\,and \hfill \\ \Gamma \left( y \right)=\mathop \smallint \limits_{0}^{\infty } {t^{y - 1}}{e^{ - 1}}dt \hfill \\ \end{gathered}$$

Example: packet drop = 0.970614, delay = 0.019489, RP = 0.357069, Success rate = 1, *N* = 635, feedback = 1.

$$=\frac{{1+1+0.357069+1}}{{1+1+0.357069+0.970614+0.019489+0.00157+2}}=0.52~$$

**Fig. 3:**
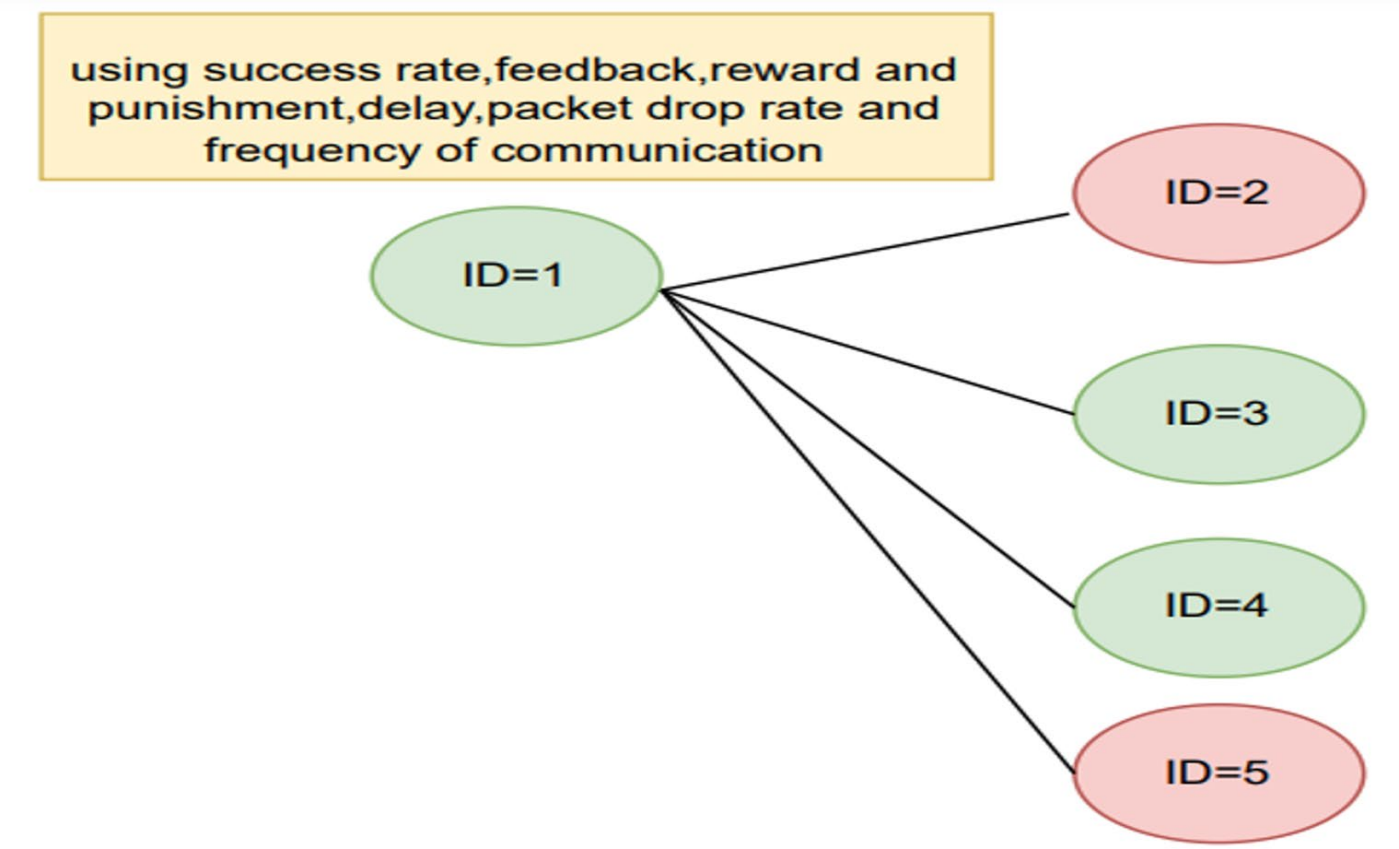
Trustworthiness evaluation between nodes.

Similarly, for each node transaction, the trustworthiness is evaluated using Eq. (9). Now, the resultant vector is the trustworthiness values for each transaction based on the node’s good and bad behaviours. The process is shown in Fig. 3, where node with id 1 is evaluating id 2, 3, 4 and 5 using aforementioned Eq. (9). It is in contrast to other works where only single parameters is used to evaluate the trustworthiness of a node.

Step 3: Trust prediction module

In literature, the use of neural networks to address nonlinear classification and regression problems is explored and demonstrated through various experiments. However, Artificial Neural Networks (ANNs) face several issues that make them unsuitable for resource-constrained IoT environments. One of the most common challenges is the vanishing gradient problem, which arises from improper weight assignments. Moreover, traditional ANNs are computationally intensive. To address this, Huang and his colleagues proposed a Single Layer Feed Forward neural network, also known as ELM, which does not use the gradient descent method for weight updates. Instead, it relies on random fixed weight assignments, integrating artificial intelligence techniques^24^. Unlike other Machine Learning and Deep Learning models KELM is lightweight in terms of speed, simple structure and low computational cost. It uses single step linear algebra for modelling the data. Therefore, in solving simple linear system equations it is fast and efficient. In the literature, KELM is used for complex tasks like hyperspectral image classification^25^, financial modelling^26^, and disease detection. It showed good performance on all of the mentioned applications, and this is the real motivation behind considering the Kernel Extreme Learning Machine (KELM) for this work. Therefore, in this work, KELM (Kernel ELM) is employed to predict the node’s trust value. It involves Moore–Penrose (MP) generalized inverse to evaluate the output of hidden neurons as shown below as H. Here python library scipy.linalg is used to evaluate the MP inverse. Additionally, the model is trained with optimal hyperparameters as Hidden units: 996, activation function: Relu, C value: 0.72 and weight: normal. The optimal parameters are evaluated using optuna framework^25,26^. In the literature, several hyperparameter optimization techniques such as Genetic Algorithms^27^ and other metaheuristic-based methods are widely used. However, these approaches generally follow a define-and-run paradigm, where the entire search space must be specified before execution. In contrast, Optuna adopts a define-by-run paradigm, enabling the search space to be constructed dynamically during execution. This flexibility makes Optuna more adaptive, cost-effective, user-friendly, efficient, and suitable for dynamic optimization scenarios.

The step of trust prediction is missing from the existing benchmarked works^7,27^. They worked only on the generation of trust labels with entropy based technique and then threshold based label generation followed by misbehaviour detection. The proposed KELM is further compared with available different versions of multiple hidden layer neural networks like LSTM (Long Short Term Memory), MLP(Multi Layer Perceptron) and GRU(Gradient Recurrent Unit). It showed remarkably well results for three out of four attacks. The comparison of all the NN is done on parameters like MSE, R^2^, MAE and time. The working of trust prediction module is shown below in algorithm 1.

**Algorithm 1:**
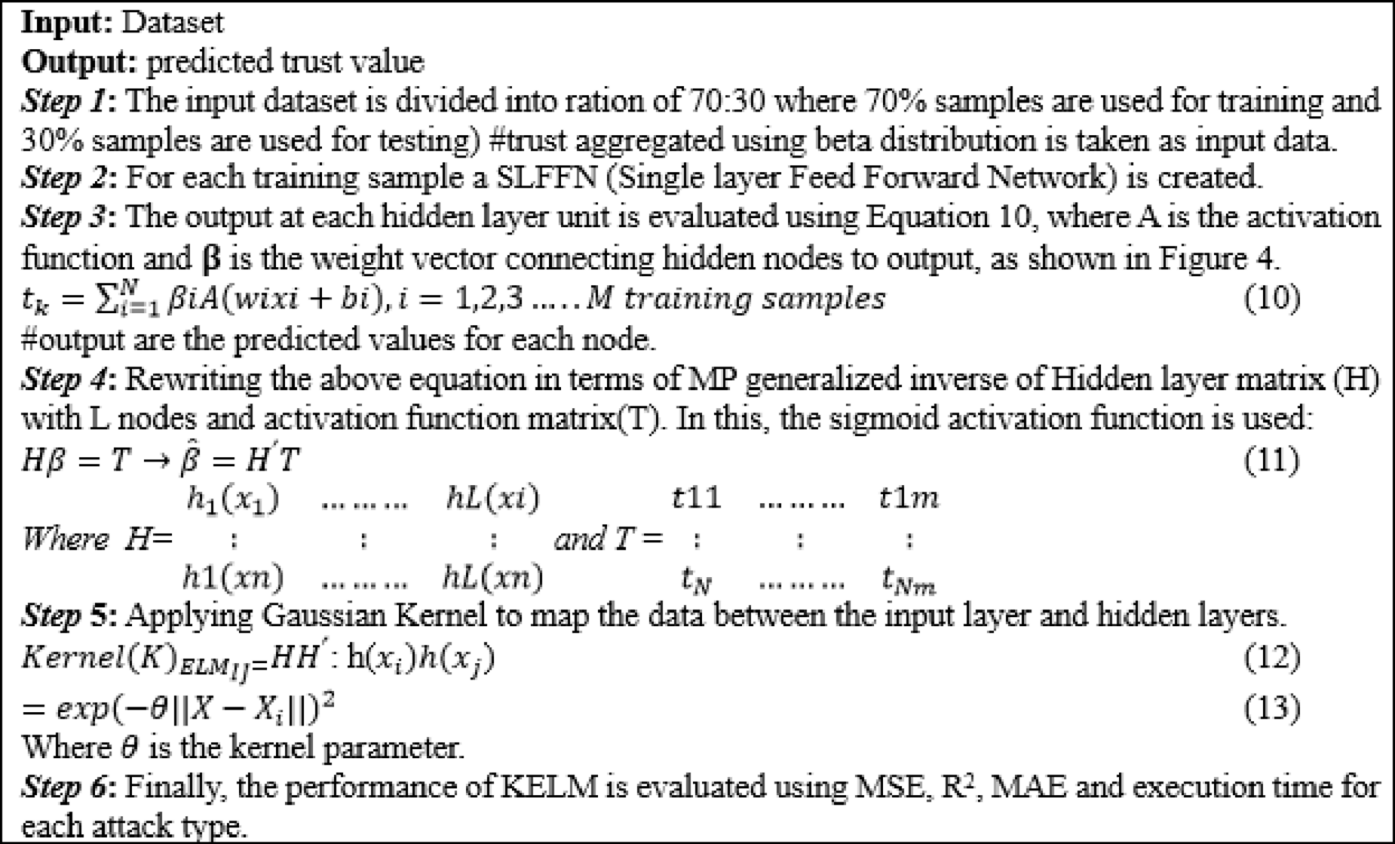
.

**Fig. 4:**
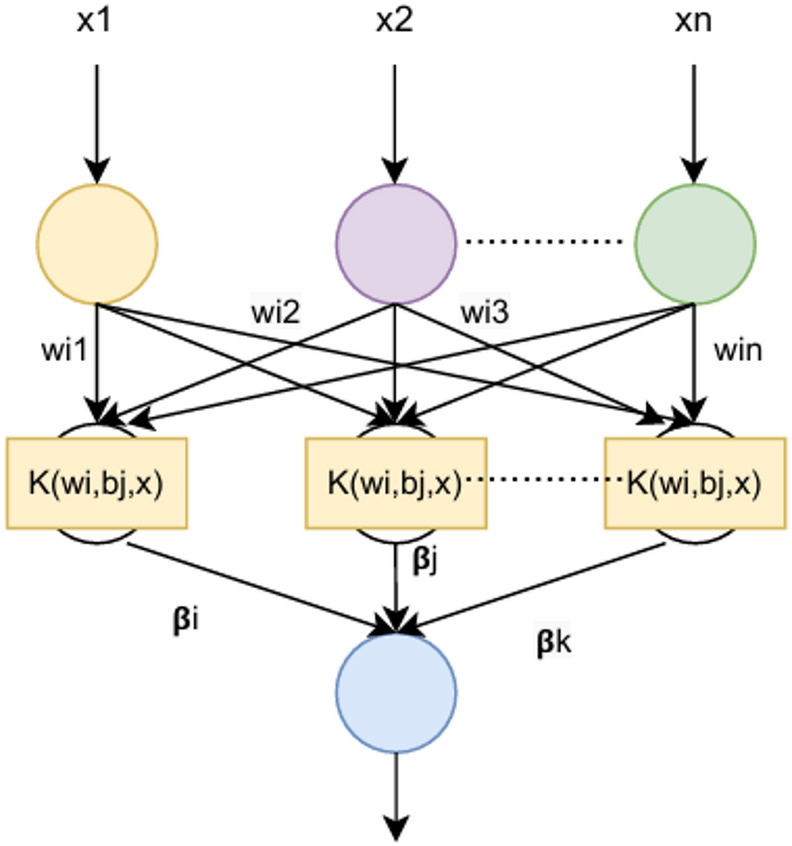
Proposed KELM.

Step 4: Attack detection module.

In the pipeline of the proposed TMS shown in Fig. 4, the Attack detection module is proceeded by the generation of labels based on the trust value predicted by KELM discussed above. For this, the K Means Clustering algorithm is employed. The working of this algorithm is shown in Algorithm 2. Using this algorithm, the Untrustworthy or “1” is the label applied to the points surrounding the cluster centroid of the untrustworthy region. On the other hand, “0” is applied to the points surrounding the cluster centroid of the trustworthy region. The process is repeated for each considered attack type. After the generation of real time labels, these labels become input for the machine learning model. The ML model analyses the Large-scale dataset given as input with ground trust values and the discovery of hidden patterns can be facilitated by these techniques, leading to more precise and flexible behaviour predictions. ML is the most powerful tool for these tasks^16^. ML models are trained on the labelled dataset for binary and multi-classification. For this, the dataset is divided into a 70:30 ratio where 70% o the total samples are used as training data and 30% are treated as test data.

**Algorithm 2:**
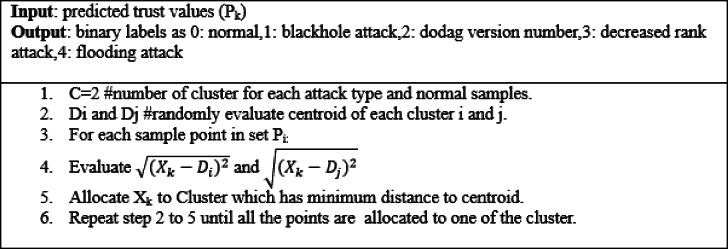
.

**Fig. 5:**
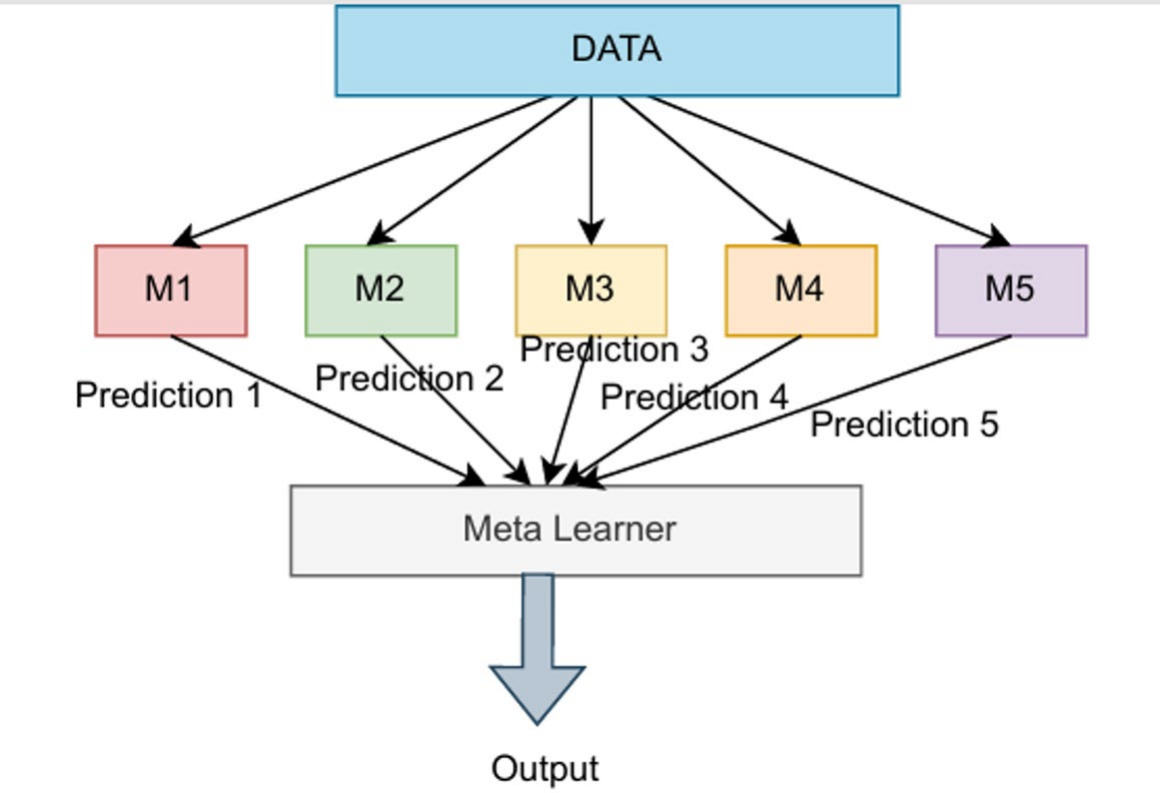
Stacked ML model architecture.

The binary classification is performed for each attack type using various ML classifiers like KNN, LR, SVM, XGBOOST, RF, DT, and SGB. The performance of each classifier is evaluated critically, and the best performing classifier is selected for multiclassification task. After this, each classifier’s performance is measured on multiclassification task (detection of each type attack). During experimentation, it was seen that the individual classifier performed poorly on this task. Therefore, a combination of 5 best classifiers is taken. This approach is considered a stacking, as shown in Fig. 5 where M1, M2, M3, M4, and M5 are selected and combined with meta classifier for accurate predictions. Additionally, cross validation for two K values, K = 5 and K = 10 is performed to prevent overfitting of the proposed model. Further, we evaluated pairwise error correlations among the constituent classifiers and observed complementary error patterns across classes. This confirms that the ensemble’s performance gains stem not only from aggregation but also from meaningful diversity among the base learners.

## Experimentation and results

### The dataset

**Fig. 6:**
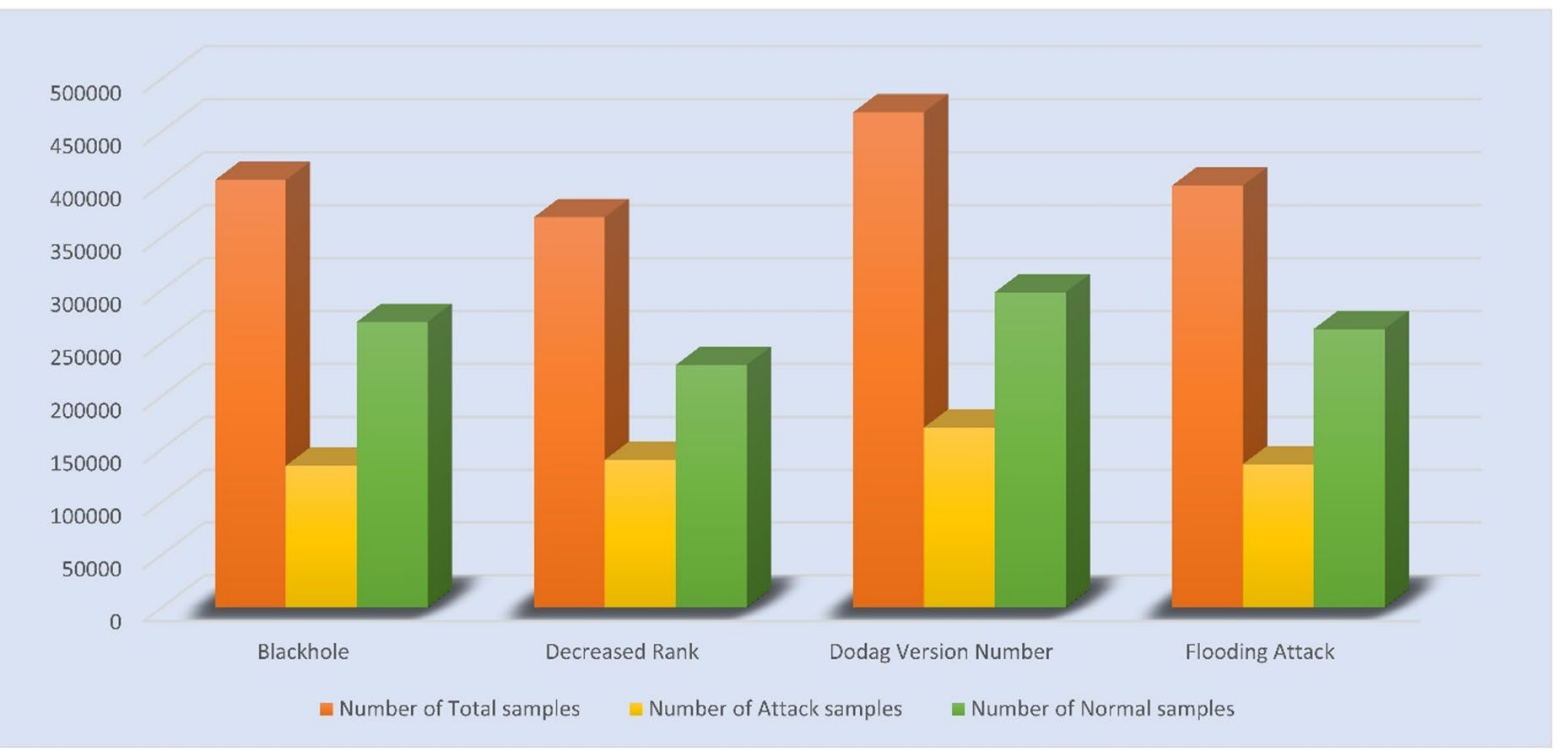
Distribution of data for each attack type along with normal samples in the ROUT-4-2023 dataset.

Finding a trust-based dataset, especially one focused on RPL-specific attacks, is difficult. To address this challenge, the benchmark dataset ROUT-4-2023, as proposed by Murat and Mehmet^29^ available at IEEE DataPort is utilized to develop the proposed system. The dataset can be accessed at “ROUT-4-2023: RPL Based Routing Attack Dataset for IoT | IEEE DataPort”. This dataset is also used in the literature to develop various intrusion detection systems (IDS)^30^. The dataset contains 18 features available in CSV format as discussed in Table 2 and the distribution of samples of each four types of attacks are shown in Fig. 6.

**Table 2:** Available features in dataset.

Feature name	Feature name	Feature name
Simulation time	Transmission rate	Transmitted packet count
Source node IP	Reception rate	Received packet count
Destination node IP	Transmission average time	Total transmission time
Packet length	Reception average time	Total reception time
Packet information	DAO packet count	DIS packet count
Category	DIO packet count	Label

The labels available in the dataset are binary (0,1), indicating anomaly or normal behaviour. This does not indicate the trustworthiness of a transaction. Therefore, these features are transformed to represent the true behaviour of a particular node (trustor) while communicating with another node (trustee). Further, the dataset contains four popular routing attacks: Blackhole, DODAG version number, decreased rank attack and flooding attacks. The distribution of samples for each type of attack and normal samples is represented in Fig. 6. All the features discussed in “Proposed data driven trust management system” are extracted from the available feature set in the dataset. The main reason for selecting this dataset is its dynamic nature, which includes trust-related attacks. Furthermore, it contains a substantial number of attack and normal samples, which aids in validating the scalability of the proposed solution.

### Model setup

This section presents the experimental information in terms of hardware and software requirements of the proposed TMS for enhancing IoT security. This work is carried out using DELL(inspiron13 5000) Laptop, with Windows 10 Enterprise 64-bit operating system installed, Intel(R)Core(TM)i5-8250U CPU @ 1.60 GHz,1.80 GHz.8.00 GB RAM. Various libraries like scikit-learn, Matplotlib, pandas and NumPy in Python programming environment (python 3.11.9) are used for data analysis and visualization. The model implementation is done using cloud based environment (google colab). Furthermore, Draw.io tool is used to create various figures for this paper.

### Machine learning algorithms

Seven Machine Learning models, such as RF, DT, XGB, SVM, LR, KNN, and SGB are used and compared during the experimentation. Additionally, a stacking model, which combines the top five best performing classifiers, is introduced for multiclass classification, as the individual classifiers mentioned above do not perform well.

- Decision Tree (DT): A classification technique that creates a tree-like structure for decision making. The tree includes decision nodes and leaf nodes. Decision nodes are further classified into sub nodes, whereas the leaf node represents the final output^30^.
- Random Forest (RF): It is an ensemble technique that combines various decision trees trained on sub samples of dataset. All the trees vote for the class of the sample, and most popular class is considered the sample’s final class^31^. It is better than a single decision tree regarding improved accuracy and overfitting.
- Extreme gradient boosting (XGB): It is a supervised algorithm that uses gradient boosted decision trees for classification. It uses a set of weak learners to create a strong predictive model where the previous learner’s result is sequentially given to the next learner. This classifier works on learning from past mistakes^32^.
- Stochastic Gradient Boosting (SGB): It is an improvement over the gradient boosting approach. The classifier divides the dataset into two sub-data sets, where one set is used for training the base learner and the other is used to test, improving the boosting model’s generalizability^33^.
- Support Vector Machine (SVM): It is a supervised algorithm that classifies data into two or more classes. It creates a hyperplane with maximum margin between two distinct classes^34^.
- Logistic Regressor (LR): This classifier works by considering the maximum likelihood estimation of an event. It uses a sigmoidal function to evaluate the probability and classify the sample based on its probability in 0 or 1 class^35^.
- K Nearest Neighbour (KNN): It is a nonparametric learning algorithm. The class of a sample using KNN is decided by considering the class of the nearest neighbour to the sample^36^.
- Stacked model: It is the combination of five best performing classifiers among all the classifiers discussed above. The details of hyperparameters of each classifier used in this study is shown in Table 3.

**Table 3:** List of hyperparameters considered in this study.

Model	Hyperparameters
DT	n_estimators(m), max_depth(n), Criterion(p)
RF	n_estimators(m), max_depth(n), Criterion(p)
XGB	n_estimators(m), max_depth(n), Subsample(o), Learning rate
SGB	n_estimators, max_depth’, Subsample(o), Learning rate
SVM	Penalty, loss, C(regularization)
LR	max_iteration, penalty
KNN	n_neighbors, weights, metric

### Performance metrics used in this study

**Table:** 

MSE	$\:\frac{1}{N}{\sum\:_{i=1}^{N}({y}_{i}-{\widehat{y}}_{i})}^{2}$	It is the mean difference between the actual and predicted values
R^2^	$\:1-\frac{{\sum\:_{i=1}^{N}({y}_{i}-{\widehat{y}}_{i})}^{2}}{{\sum\:_{i=1}^{N}({y}_{i}-{\stackrel{-}{y}}_{i})}^{2}}$	It is the representation of goodness of fit, how well the actual and predicted values fit
MAE	$\:\frac{\sum\:_{i=1}^{n}\left|{y}_{i}-{x}_{i}\right|}{n}$	It determines the average of the absolute errors between the predicted and actual values
Accuracy (Trian and test accuracy)	$\:\frac{TP+TN}{TP+TN+FP+FN}$	It is the ratio of samples correctly classified and the total number of samples
Precision	$\:\frac{TP}{TP+FP}$	It is defined as the ratio of the number of attack samples detected correctly and the total number of actual attack samples
Recall	$\:\frac{TP}{TP+FN}$	It is defined as the ability of a model to classify all attack samples correctly
F-Score	$$\:\frac{2*Precision*Recall}{Precision+Recall}$$	It is a balancing factor of both precision and recall
Time in execution(s)	time.time-begin_time	Time between the start and end of the execution of the proposed model

### Results and analysis

**Fig. 7:**
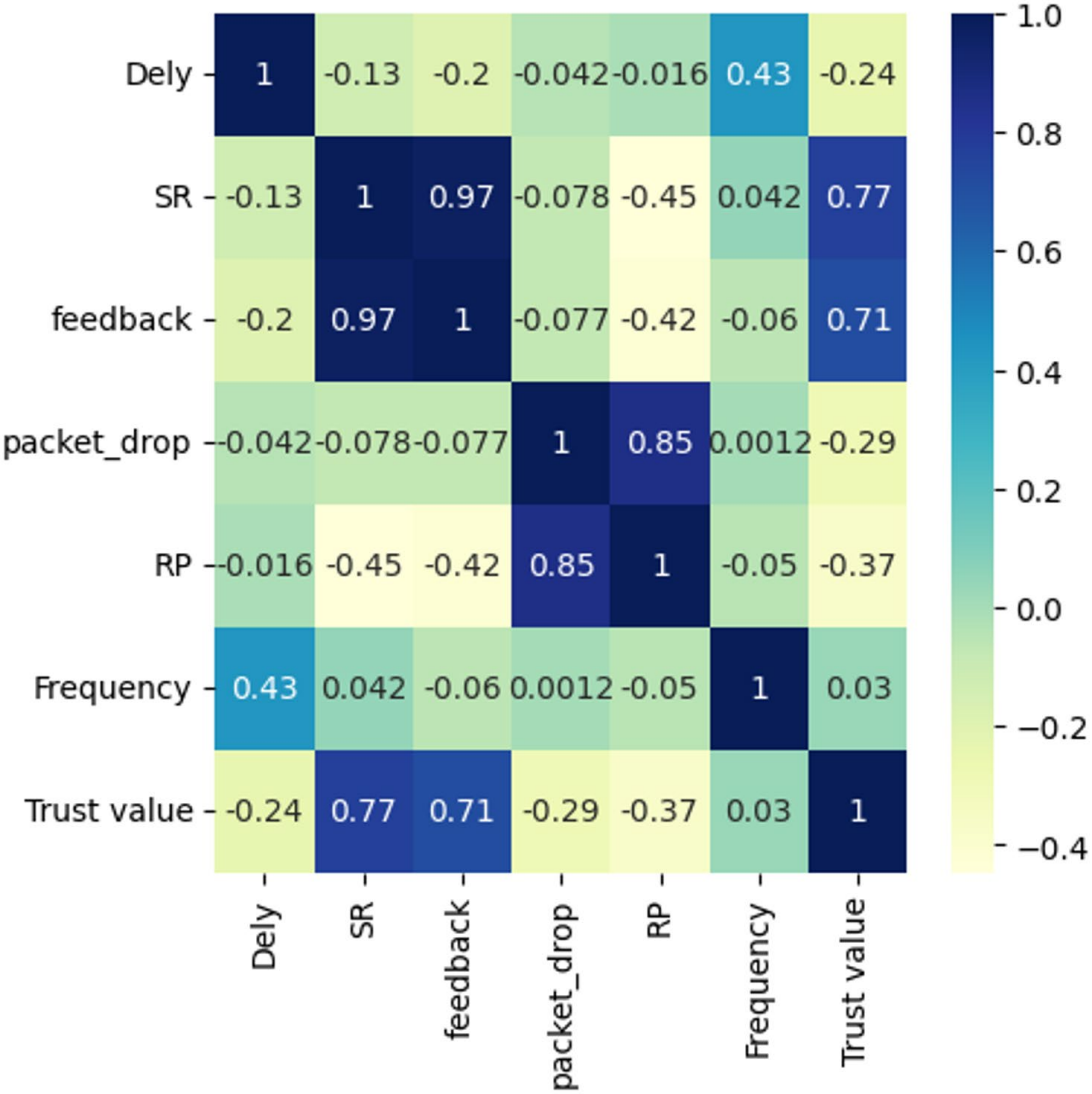
Relation between each pair of proposed trust metrics.

**Fig. 8:**
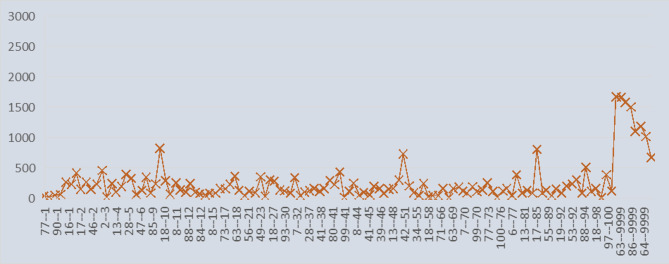
Frequency of communications between source and destination.

This section discusses the results and visualization of each module of the proposed TMS. The six features extracted from the dataset described in Sect.  3 are delay, success rate, feedback, packet drop rate, Reward and punishment (RP) and frequency of communication. The relationship between each extracted feature and the evaluated trust value (target) is shown in Fig. 7. This indicates that when SR is high trust value is also high showing positive correlation with value 0.77, feedback is also positively correlated with trust value (good behaviours) showing 0.71 corelation value, and Packet drop, delay and RP are negatively correlated with trust value showing high negative value. Additionally, a small positive correlation is visible between the frequency of communication and trust value. Additionally, the frequency of communication depicts the number of times a node communicates with other nodes. Likewise, as shown in Fig. 8, Node no. 63 and 9999 have the highest number of communications.

**Fig. 9:**
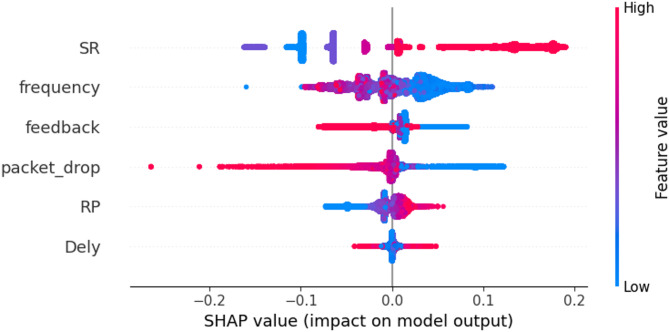
SHAP analysis of each predictor.

In addition to correlation, the following SHAP (Shapley Additive Explanations) analysis is also performed to show the contribution of each indicator towards trust prediction accuracy. This analysis helps in understanding the local as well as global role of each indicators. It shows how much a particular feature have contributed towards a particular outcome. Therefore, it is visible form Fig. 9 that SR has the highest influence on the trust prediction followed by frequency, packet drop, RP, feedback and delay. As a result, SR contributes up to ~ 20% change in model output, while Delay contributes ~ 3%. Here, a trustworthy node is defined as a node having high success rate, drops few packets, communicates at reasonable frequency, generates few negative feedback events and show low delay.

### Trust value prediction results

**Fig. 10:**
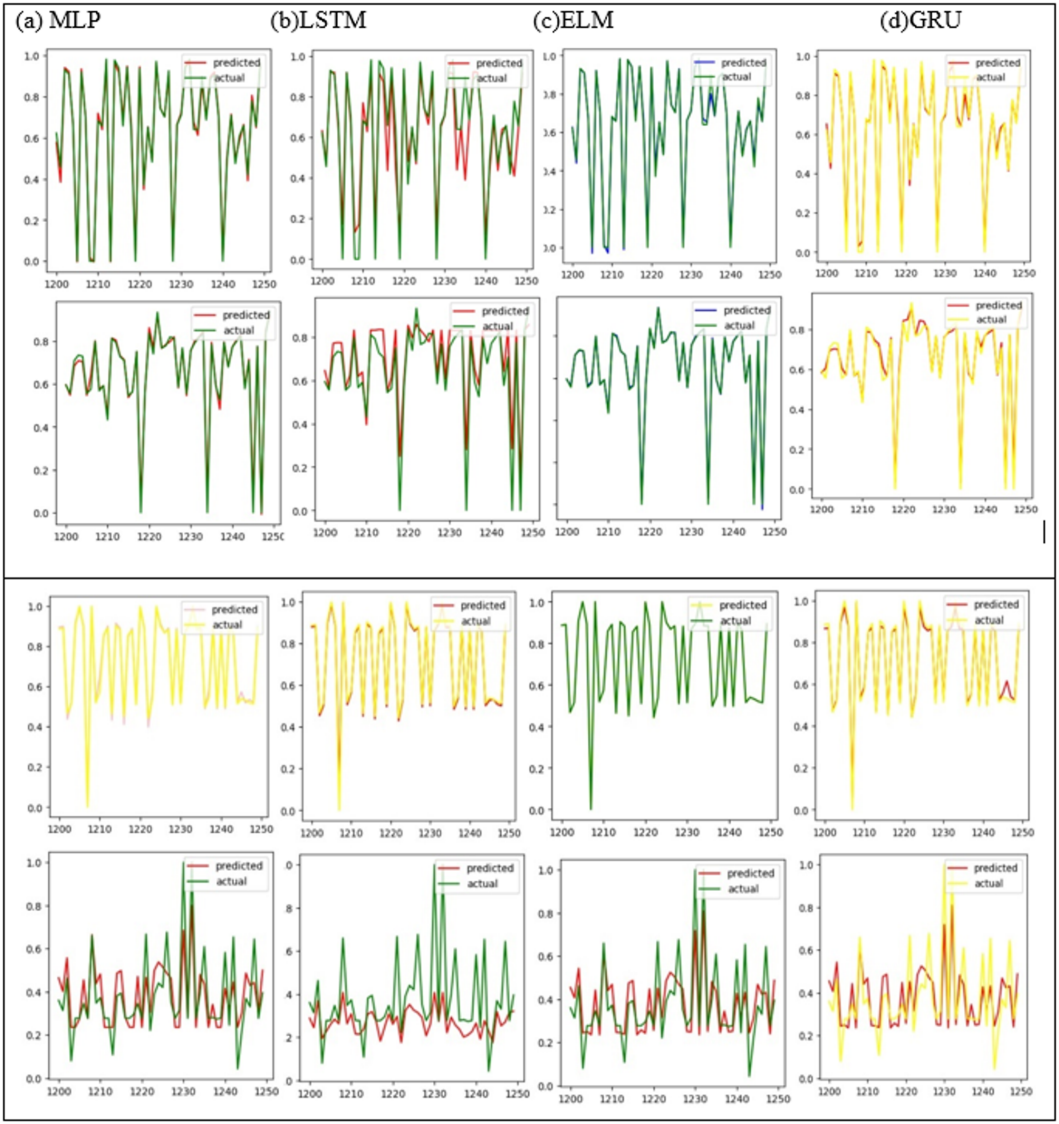
Prediction results for each Predictor MLP (1st column ), LSTM (2nd column), KELM (3rd column), GRU (4th column) for each type of attacks starting form blackhole (1st row), decreased rank (2nd row), flooding (3rd row) and version number (4th row).

The results of trust prediction module for real time trust value prediction of nodes are discussed in this section. KELM, as shown above is applied to four attack types, flooding, blackhole, rank and version number attack. For each attack type, the performance of four main Neural Networks (NN) types are compared i.e., GRU, LSTM, KELM and MLP. These NN differ in internal architecture and working. For each attack type the dataset is divided into 70:30 ratio that is 70% dataset is used for training and 30% data is used for testing. A combination of different scales of datasets, like 60:40 (train: test) and 50:50 (train: test), are also tested to test the overfitting and flexibility of models, and we get the same results for all the combinations. All the NNs are compared for each attack type based on MSE, R^2^, MAE, and time as shown in Tables 4, 5, 6 and 7 .During experimentation, it was seen that a simple KELM, which is a single layer feed forward network, performs well for 3 out of 4 attack types, whereas LSTM, which is more complex than the KELM performs worst for finding the dependencies in the features and target. Each NN is compared with similar environmental conditions considering the basic structure of a NN. Like for MLP, the number of minimum layers is 3 which shows the best results, for LSTM also we have 3 layers and for GRU as well. They are also checked with single layer architecture but didn’t perform well. Therefore, the best performance are hereby reported. The performance for trust predictions based on the considered features are compared with similar work^16^ where the best MSE value is 0.008 with LSTM but in our case, the best MSE value attained by a simple KELM is 0.0000303 for flooding attacks. This is due to the simple and relevant representation of features pertaining to the target that a simple KELM can find the relation and predict better values. Moreover, R^2^, also known as the goodness of fit, indicates that the value closer to 1 represents the best fit by the model. Here, KELM show maximum value of R^2^ as 0.999, whereas the best value of R^2^ in previous work^16^ is 0.88, which shows that the predicted and actual values of the proposed KLEM are almost the same. The predictions and actual trust values are plotted and shown in Fig. 10 for each attack. The plots represent the closeness between actual and predicted values by the considered model. It shows the best performance for flooding attacks in terms of low MSE, high R^2^, low MAE and less time. For version number, it shows low MSE, moderate MAE, moderate R^2^ and moderate time, whereas for rank, it shows low MSE, high R^2^, low MAE and less time. Additionally, the minimum time KELM takes to predict the trust value is just 0.0257s, which shows the speed of prediction of a KELM model. This indicates the advantages of KLEM over other considered Neural Networks.

**Table 4:** Prediction performance of neural networks for rank attack.

Rank				
	MSE	R2	Time	MAE
GRU	0.00015	99.12	129 s	0.0085
LSTM	0.0002	98.51	347 s	0.0075
ELM	4.67E−07	99.99	27.15 s	0.0003
MLP	8.88E−05	99.48	531 s	0.0066

**Table 5:** Prediction performance of neural networks for blackhole attack.

Blackhole				
	MSE	R2	Time	MAE
GRU	0.0006	0.99	1982 s	0.019
LSTM	0.0087	0.8686	2361 s	0.05
ELM	0.0003	0.995	1781 s	0.0085
MLP	0.0038	0.942	2635 s	0.044

**Table 6:** Prediction performance of neural networks for version number attack.

Version number attack				
	MSE	R2	Time	MAE
GRU	0.0126	0.554	619 s	0.093
LSTM	0.034	− 0.201	422 s	0.142
ELM	0.0115	0.6	560 s	0.09
MLP	0.012	0.545	606 s	0.096

**Table 7:** Prediction performance of neural networks for flooding attack.

Flooding attack				
	MSE	R2	Time	MAE
GRU	0.0006	0.978	2505 s	0.017
LSTM	0.005	0.814	2256 s	0.058
ELM	3.03E−05	0.9989	0.0257	0.002
MLP	0.0004	0.9835	1969	0.013

### Label generation results

**Fig. 11:**
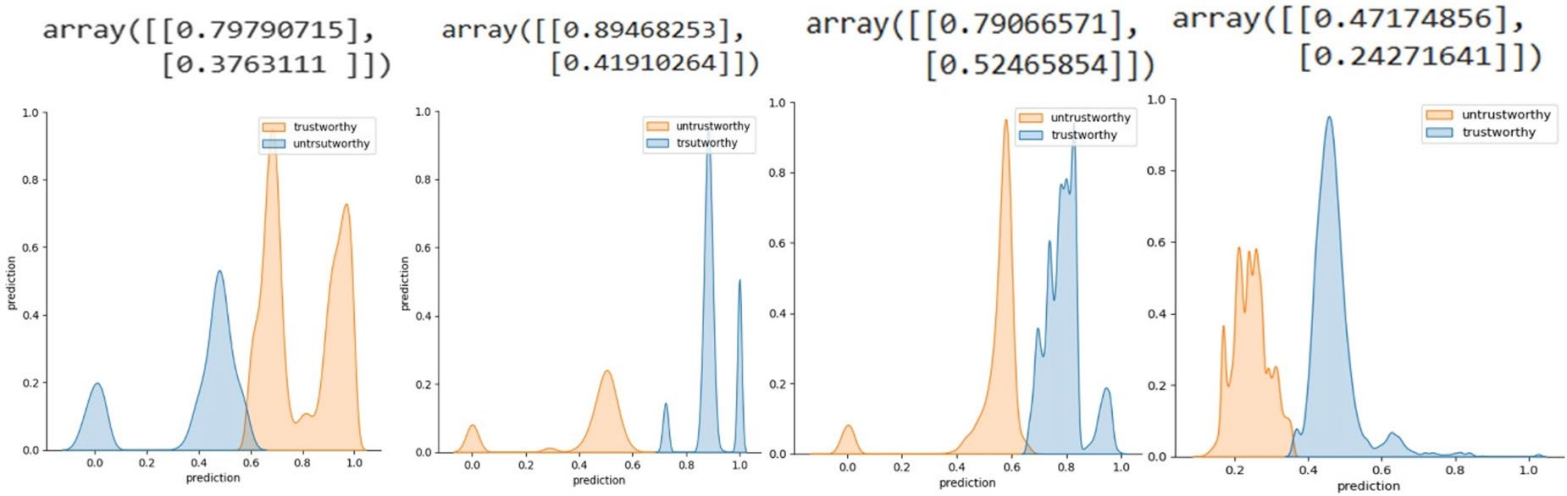
Clusters showing dynamic threshold for each attack type (**a**) Blackhole. (**b**) Decreased rank. (**c**) Flooding. (**d**) Version number.

Once the trust values are accurately predicted, they are utilized to generate a trustworthy or untrustworthy label through Algorithm 2. For each of the attack types, algorithm 2 is run, and a dynamic threshold can be seen. This shows the dynamicity of the proposed TMS framework. Therefore, it can handle any type of internal attack by using a dynamic threshold. As shown in Fig. 11, it is visible that two thresholds are generated for each attack. One threshold shows a trustworthy threshold, which indicates that the predicted trust values close to this will be considered trustworthy, and another threshold shows the threshold for untrustworthy predicted trust values, the values close to this threshold will be considered as untrustworthy.

**Fig. 12:**
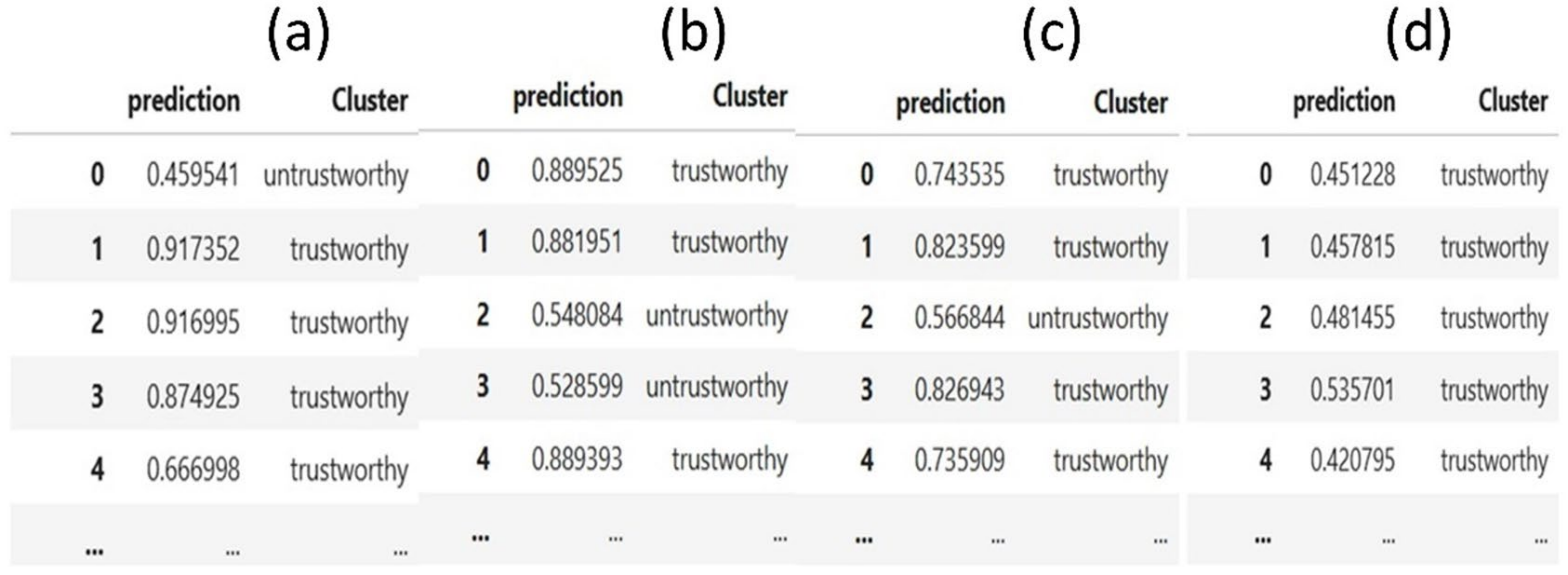
Results on dataset for each attack type. (**a**) Blackhole. (**b**) Decreased rank. (**c**) Flooding. (**d**) Version number.

Here, for blackhole attack values near 0.79790715 are considered as trustworthy, whereas values near 0.3763111 are treated as untrustworthy. Similarly, for rank attack the trustworthiness is quite high, the trust values close to 0.89468253 are considered as trustworthy whereas the values near 0.41910256 are treated as untrustworthy. Similarly, for flooding attacks trustworthiness evaluated as 0.79066571 are considered as trustworthy whereas 0.52465854 are treated as untrustworthy. Further, for version number trust value of a node close to 0.4717856 is treated as trustworthy whereas 0.24271641 is treated as untrustworthy. It can be seen that creation of clear boundary for version number is quite difficult as the real and attacking behaviours are quite similar and hence low distance between two clusters. The predicted trust values for each attack using python notebook are displayed in Fig. 12. In contrast to using one single static threshold for attack detection, a dynamic threshold which is robust and generalized approach is proposed in this work.

### Attack resilience and robustness results

**Fig. 13:**
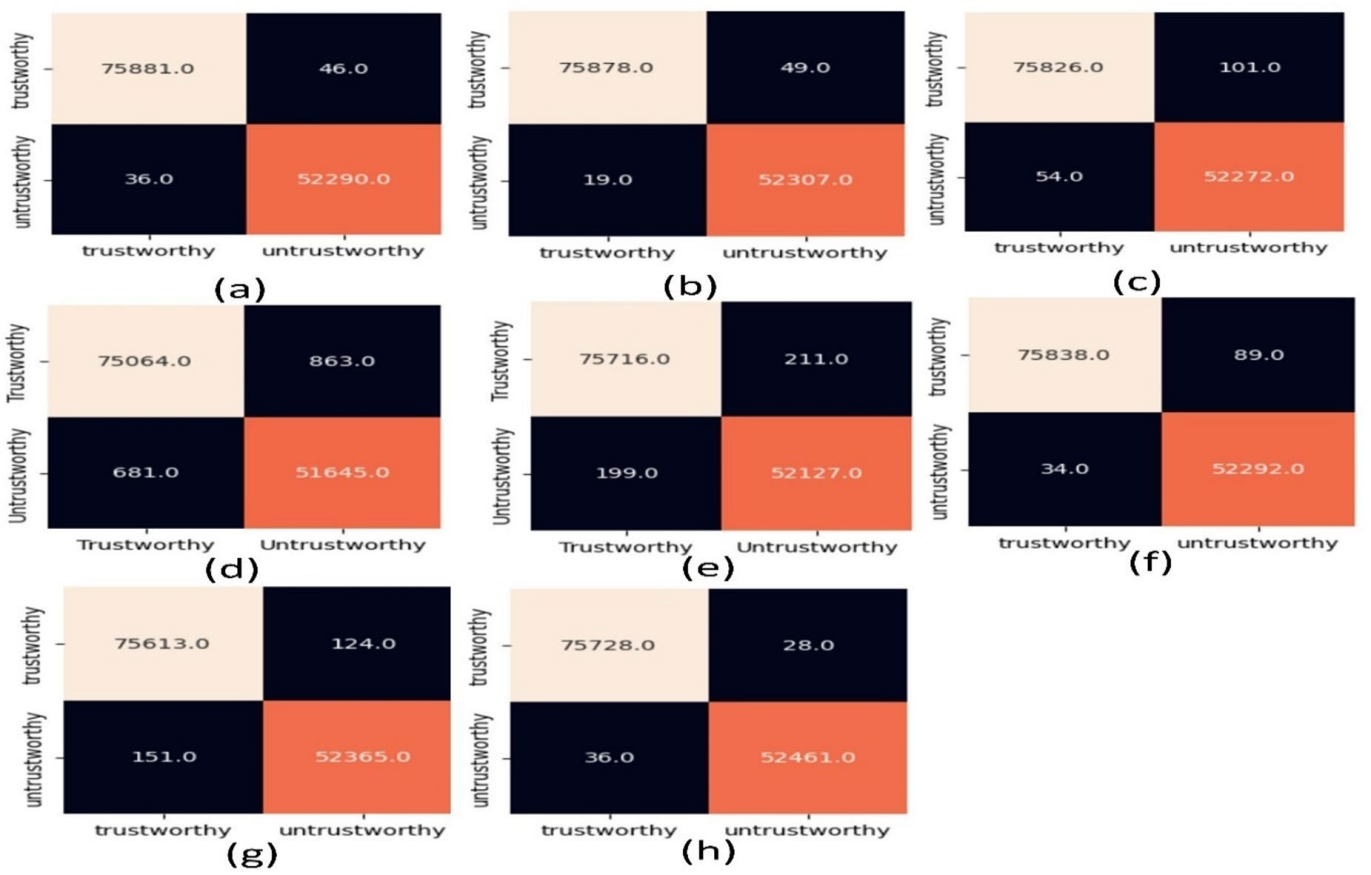
Confusion matrix representing binary classification performance of: (**a**) Decision Tree. (**b**) Random Forest. (**c**) K Nearest Neighbour. (**d**) Logistic Regressor. (**e**) SVM. (**f**) XGB. (**g**) SGB. (**h**) Stacking.

**Fig. 14:**
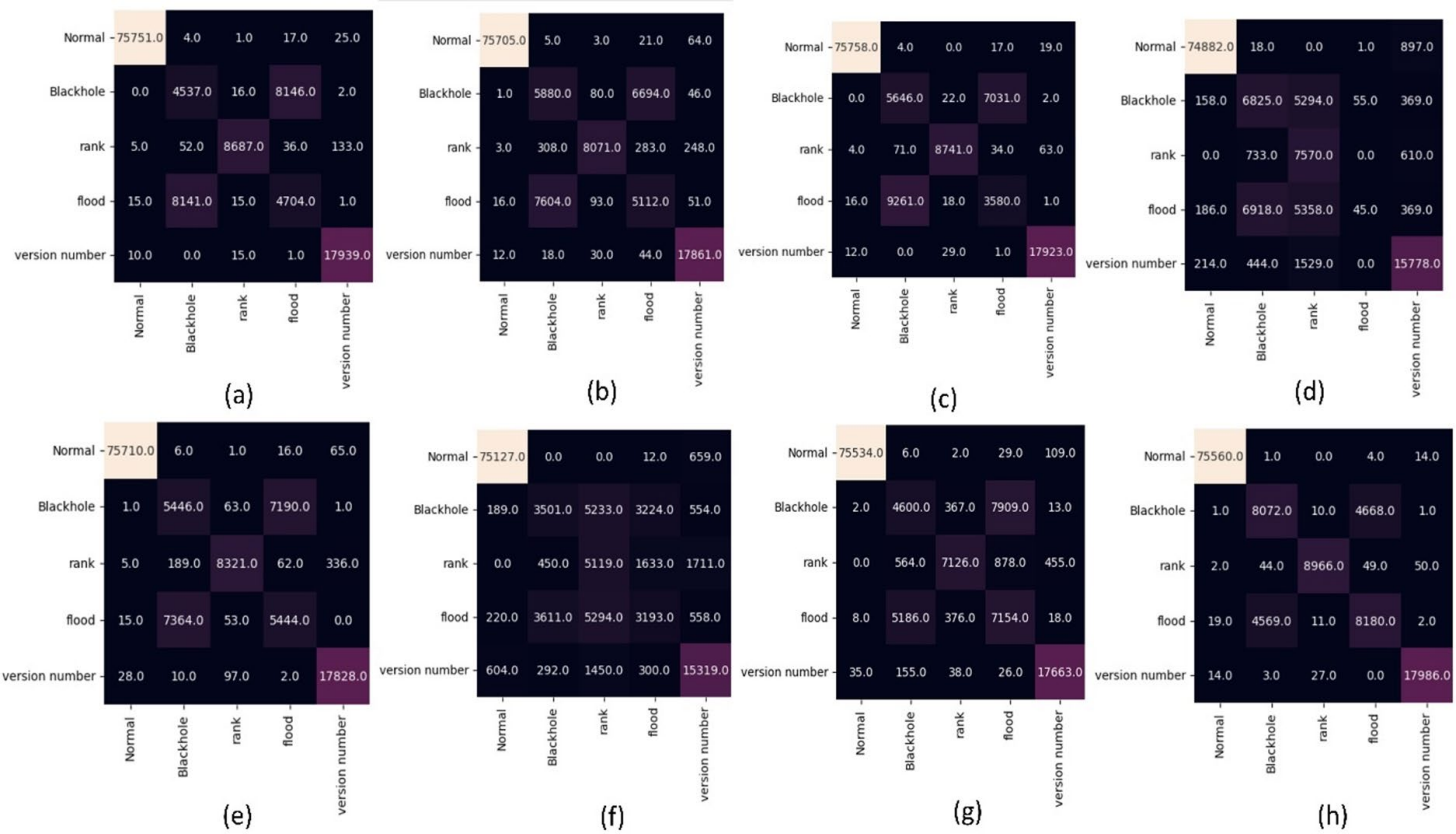
Confusion matrix representing multi classification performance of: (**a**) Decision Tree, (**b**) Random Forest, (**c**) K Nearest Neighbour, (**d**) Logistic Regressor, (**e**) SVM, (**f**) XGB, (**g**) SGB, (**h**) Stacking.

The model trained using on the dataset is evaluated for detection of binary (anomaly detection) and multiclassification (attack detection).The performance of each classifier for both the considered task is depicted in Tables 8 and 9. The stacking model is the combination of 5 best classifier that perform well on most of the performance indicators as compared to individual classifier as shown in tables. The highest accuracy reached by the stacking technique for binary classification is 99.94% as compared to RF which shows best among all with 99.95 and precision as 99.91, similar to DT. The best recall as 99.95 and f score as 99.93 is shown by RF which is similar to stacking. Furthermore, a confusion matrix is a table showing performance of a model based on actual and predicted outcomes as shown in Figs. 13 and 14. In Fig. 13 RF got confused among 68 samples whereas stacking got confused among 64 samples. Therefore, for anomaly detection also the stacking technique showed better results as individual classifier.

Considering attack detection the individual classifier showed a performance with major confusions in classifying attacks and normal samples. Hence, showing high false alarms. Due to this Stacking of best performing model is proposed which performs better in attack detection, individual classifiers got confused between attack types and normal samples, whereas stacking technique got confused among types of attacks but not attacks and normal samples as shown in Fig. 13. Hence, showing less False alarms. The performance of each classifier and stacking for attack detection based on accuracy, precision, recall and f score is shown in Table 9.

**Fig. 15:**
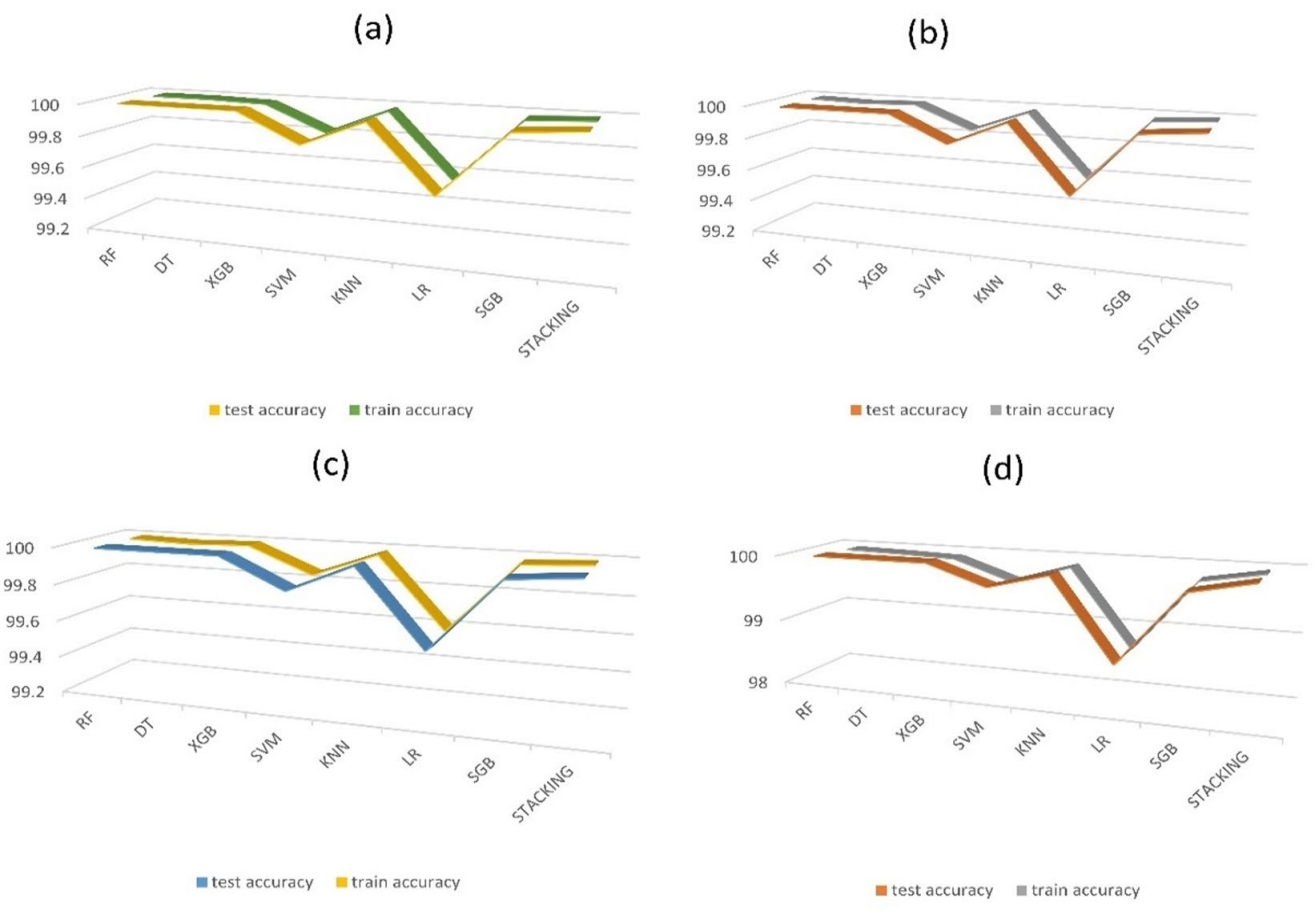
Test and Train accuracy of each classifier for (**a**) 25% dataset, (**b**) 50% dataset, (**c**) 75% dataset, (**d**) 100% dataset.

Further, the performance of all the techniques are validated in terms of test and train accuracy for varying size of dataset (25%, 50%, 75% and 100%), it can be seen in Fig. 15 that stacking technique outperforms all the other individual classifier on each dataset sizes.

**Table 8:** Performance of each classifier for anomaly detection (binary classification).

	RF	DT	XGB	SVM	KNN	LR	SGB	Stacking
Accuracy	99.95	99.93	99.9	99.6	99.87	98.7	99.78	99.94
Precision	99.9	99.91	99.83	99.5	99.8	98.3	99.9	99.91
Recall	99.96	99.93	99.93	99.6	99.8	98.6	99.93	99.95
F-Score	99.93	99.92	99.88	99.61	99.8	98.5	99.91	99.93

**Table 9:** Performance of each classifier for attack detection (multi classification).

	RF	DT	XGB	SVM	KNN	LR	SGB	Stacking
Accuracy	87.06	87.06	87.81	81.94	87.91	79.73	87.53	92.7
Precision	87.15	87.06	87.97	83.3	88.6	80.2	87.6	92.8
Recall	87.13	87.12	87.8	82.1	88.1	80.1	87.53	92.7
F-Score	87.14	87.09	87.89	82.81	88.37	80.15	87.56	92.75

## Comparison with state of the arts techniques

**Fig. 16:**
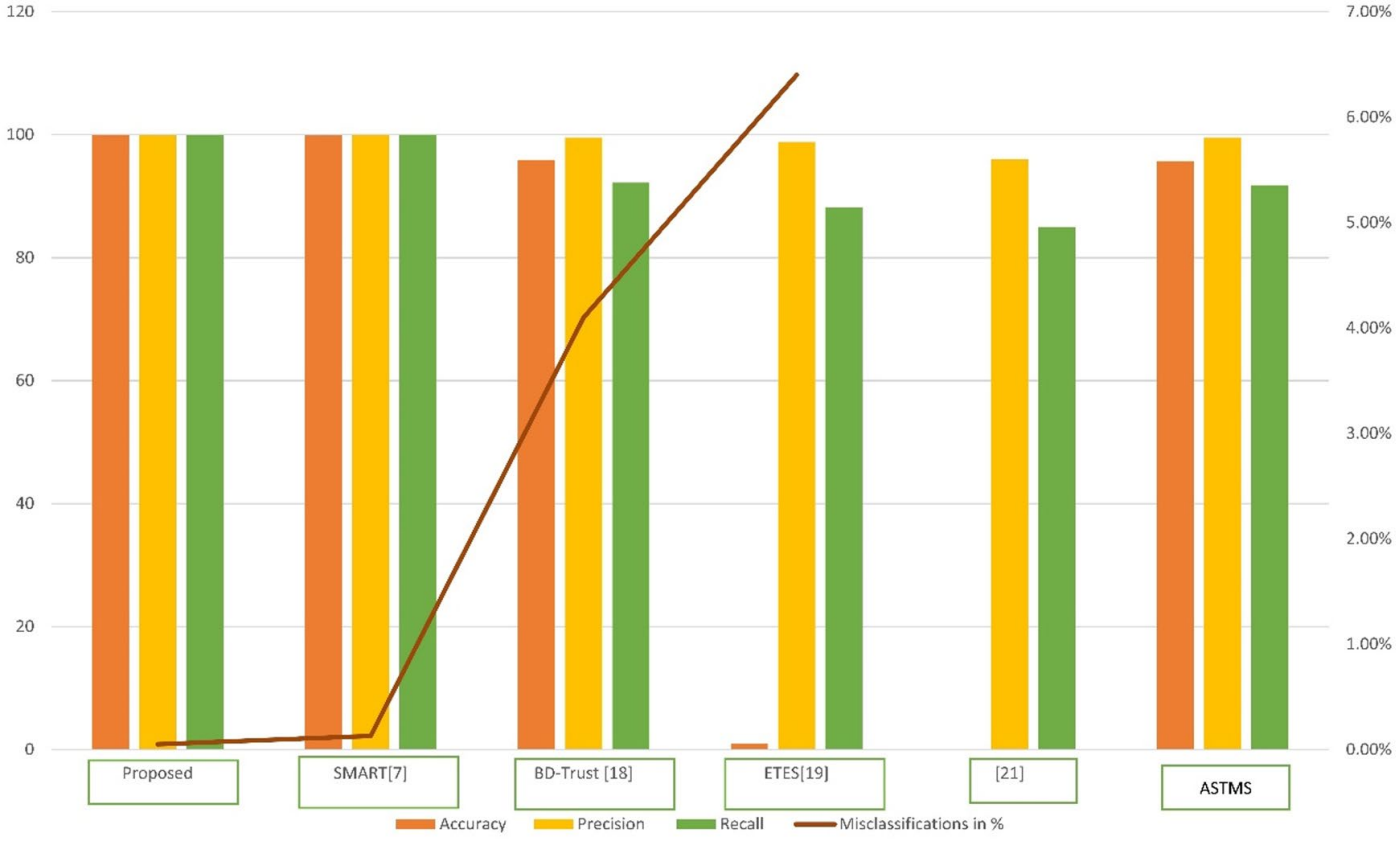
Performance comparison among state of the art TMS.

The proposed model’s performance is compared with the existing TMS to validate our TMS’s performance. The performance metrics considered for comparison are accuracy, precision, recall and misclassification rate. As shown in Fig. 16, our proposed model outperforms state of the art TMS. ASTMS proposed in^37^ shows accuracy of 95.7%, precision of 99.5%, recall of 91.8% and misclassification rate of 4.3%, whereas the TMS BD-Trust proposed in^18^ shows better performance than^37^ with accuracy of 95.9%, precision as 99.5%, recall as 92.2% and misclassification rate as 4.1%. Another popular TMS ETES proposed in^38^ with a high misclassification rate of 6.4%. This TMS show accuracy of 93.6%, a precision of 98.8% and a recall of 88.2%. The TMS proposed in^20^ shows precision of 96%, Recall of 85% and f1 score of 87%. Another popular TMS proposed in^7^ perform better than the above-mentioned TMS with a high accuracy of 98.97%, precision of 99.93%, recall of exactly 1 and misclassification rate of 0.13%. These methods are validated on a very small dataset compared to ours. Our proposed TMS show comparative performance in most metrics but outperforms^7^ in misdetection rate with just 0.05%. Further, the proposed TMS show 99.95% accuracy, 99.9% precision, 99.96(almost 1) % recall and only 0.05%misclassification rate.

## Conclusion

The Internet of Things (IoT) applications are extensive, covering a wide range of devices within the network. However, security and privacy remain the primary focus of researchers in IoT. Trust Management Systems (TMS) applications for access control, authentication, and attack prevention are well-documented in the literature. Most trust-based models focus on detecting either On-Off attacks or selective forwarding attacks, with very little research addressing RPL specific attacks. Therefore, this paper proposes a lightweight and real-time TMS for securing IoT networks against four types of RPL specific attacks: blackhole, rank, flood and version number. We introduce a novel set of trust indicators, including direct, indirect trust and socialness, to predict real time trust values of each node and restrict further communication. The proposed TMS operates in a pipeline with four key modules: trust indicator selection, trust aggregation, trust prediction, and attack detection. Further, we employ a lightweight version of the traditional neural network, KELM, for accurate and robust real time trust prediction. During the experimentation, it is seen that KELM demonstrates minimal Mean Squared Error (MSE) of just 0.0000303 as compared to other types of neural networks. This indicates a very close prediction of actual behaviour of the node. Employing the predicted trust value a number of machine learning models are compared, like RF, DT, XGB, SVM, KNN, LR and SGB, to classify trustworthy and untrustworthy transactions. For binary classification RF performs best among all the considered classifiers and got confused with 68 samples. Meanwhile, all the considered classifiers perform poorly for multiclassification (attack type detection), with an average accuracy of 87.81%. Hence, to further improve the performance a stacked model is proposed, combining five best performing classifiers, The stacked model showed improved performance of 92.7% accuracy on multiclassification and 99.96% for binary classification. Further, the performance of the proposed stacked model is tested on different data sizes (25%, 50%, 75%, 100%), where it maintains consistent performance across both training and test sets, showing nearly identical accuracy levels. Therefore, we recommend a stacked model in case IoT networks are vulnerable to multiple routing attacks. In the future, we aim to incorporate additional features to enhance system performance. The authors also intend to explore federated learning for developing trust based security frameworks for IoT.

## Data Availability

The datasets generated during and/or analysed during the current study are available in the IEEE DataPort repository at “ROUT-4-2023: RPL Based Routing Attack Dataset for IoT | IEEE DataPort”.

## Code availability

himanityagi006/STEM: code_implementation.
